# Ceritinib (LDK378) prevents bone loss *via* suppressing Akt and NF-κB-induced osteoclast formation

**DOI:** 10.3389/fendo.2022.939959

**Published:** 2022-11-08

**Authors:** Wenxin He, Xiankun Cao, Keyu Kong, Kewei Rong, Shuai Han, An Qin

**Affiliations:** ^1^ Shanghai Institute of Hematology, State Key Laboratory of Medical Genomics, National Research Center for Translational Medicine at Shanghai, Ruijin Hospital Affiliated to Shanghai Jiao Tong University School of Medicine, Shanghai, China; ^2^ Centre National de la Recherche Scientifique–Laboratoire International Associé (CNRS-LIA) Hematology and Cancer, Sino-French Research Center for Life Sciences and Genomics, Ruijin Hospital Affiliated to Shanghai Jiao Tong University School of Medicine, Shanghai, China; ^3^ Shanghai Key Laboratory of Orthopaedic Implants, Department of Orthopaedics, Shanghai Ninth People’s Hospital, Shanghai Jiao Tong University School of Medicine, Shanghai, China; ^4^ Guangxi Key Laboratory of Regenerative Medicine, Guangxi Medical University, Nanning, Guangxi, China

**Keywords:** osteoclast, ceritinib, LDK378, Akt, p65

## Abstract

**Background:**

Ceritinib is used for the treatment of patients with anaplastic lymphoma kinase (ALK)-rearranged non-small cell lung cancer (NSCLC), who are at the risk of developing bone metastasis. During bone metastasis, tumor cells release factors that induce osteoclast formation, resulting in osteolysis. However, the effect of ceritinib on osteoclast formation remains unclear.

**Methods:**

Osteoclastogenesis was induced to assess the effect of ceritinib on osteoclast formation and osteoclast-specific gene expression. Western blotting was used to examine the molecular mechanisms underlying the effect of ceritinib on osteoclast differentiation. An *in vivo* ovariectomized mouse model was established to validate the effect of ceritinib in suppressing osteoclast formation and preventing bone loss.

**Results:**

The differentiation of osteoclasts and the expression of osteoclast-specific genes were inhibited upon ceritinib stimulation. Ceritinib suppressed Akt and p65 phosphorylation during the receptor activator of nuclear factor kappa-B ligand (RANKL)-induced osteoclastogenesis. The administration of ceritinib to ovariectomized mice ameliorated trabecular bone loss by inhibiting osteoclast formation.

**Conclusions:**

Ceritinib is beneficial in preventing bone loss by suppressing osteoclastic Akt and nuclear factor κB (NF-κB) signaling.

## Introduction

Ceritinib (LDK378) is a second-generation anaplastic lymphoma kinase (ALK) inhibitor (1). It exhibits robust anti-tumor efficacy in patients with ALK-rearranged non-small cell lung cancer. Generally, it increases ALK selectivity approximately 20 times more potently than the first generation ALK-targeted compound, crizotinib (2). In crizotinib-resistant mutants, ceritinib can inhibit the activity of ALK mutants, including Leu1196Met, Gly1269Ala, Ile1171Thr, and Ser1206Tyr. Therefore, ceritinib was granted accelerated approval by the FDA in April 2014, for the treatment of ALK-positive NSCLC (3, 4).

ALK is a receptor tyrosine kinase that belongs to the insulin receptor superfamily. It is activated upon ligand-induced homo-dimerization, which can subsequently activate various signaling pathways, including Janus kinase (JAK)-signal transducer and activator of transcription (STAT), phosphoinositide 3-kinase (PI3K)-Akt, and mitogen-activated protein kinase (MAPK) signaling cascades (5). Downstream PI3K-Akt activation triggers signaling cascades mediated by transcription factors such as mammalian target of rapamycin (mTOR), glycogen synthase kinase 3β (GSK3β), and forkhead box O (FOXO). ALK activity initiates the transcription of several genes, including NF-κB (6).

Approximately, 30-40% of patients with non-small cell lung cancer develop bone metastasis during the course of the disease (7). During non-small cell bone metastasis, osteolysis prevails over bone formation due to the activity of tumor-secreted factors, such as PTHrP, IL-11, IL-6, and TNF-α, which induce excessive osteoclast formation (8). In addition to bone metastasis, changes in bone mineral density also occur; moreover, according to clinical studies, the prevalence of osteoporotic vertebral fractures is 30–40% in lung cancer patients (9). Therefore, compounds, such as bisphosphonates, that can target osteoclast formation are preferred for the treatment of bone metastasis caused due to non-small cell lung cancer or to improve the bone mass in these patients (10–12).

Osteoporosis is highly prevalent worldwide with typical characteristics of low bone mineral density and high possibility of fracture. The prevalence of osteoporosis was calculated by several countries and indicated higher risks in women from 2 to 8 times one in men (13). Excessive formation and activation of osteoclasts leads to development of osteoporosis (14). Most of current clinical antiresorptive agents include estrogen; bisphosphonates (BPs); human monoclonal antibody against receptor activator of NF-κB ligand (RANKL) denosumab; selective estrogen receptor modulators (SERM) raloxifene; and strontium ranelate (SR). However, their efficacy and safety have not been established for long-term therapy completely (15). And there have been reported that BPs caused osteonecrosis of the jaw (ONJ); and alendronate causing atypical fractures (16), and estrogen replacement therapy brought concern of increased breast cancer risk (17, 18).

RANKL, the critical factor of osteoclast differentiation, downstream signaling pathways are activated including NF-κB, JNK, p38 MAPK, extracellular signal-related kinase and Akt. RANKL activates the IκB kinase (IKK) complex and thus phosphorylates NF-κB–associated IκBα to release RelA/P65 (one of the members of NF-κB family) to cytosol. Further P65 translocates to nucleus and activates downstream transcriptions (19, 20). Phosphatidylinositol 3-kinase (PI3K) and further phospholipid dependent activation of the Akt (serine/threonine kinase) mediate to suppress the apoptotic function in various cells. Increased expression levels of phospho-Akt enhanced RANKL-induced osteoclastogenesis (21, 22). Activation of Ras, Raf-1 and mitogen-activated protein kinase (MAPK) signaling cascades are essential in cell proliferation and differentiation (23, 24). And MAPK activates downstream of ERK1 (p44 MAPK) and ERK2 (p42 MAPK) to maintain osteoclast survival and polarity, and ruffled border formation (25–27).

Myostatin binds to and activates complex of ALK4/5 and strongly accelerates RANKL-mediated osteoclast formation. Meanwhile ALK inhibitory effect has been found in reducing osteoclast differentiation critically (28). Here we introduced an anti-cancer drug ceritinib (LDK378) with additional inhibiting effect on osteogenesis and potential in treating related osteoporosis. Ceritinib was reported as an inhibitor of anaplastic lymphoma kinase (ALK) (1).

A previous study reported that the first-generation ALK-targeted compound crizotinib could prevent prostate cancer-associated bone loss (29). In this study, we aimed to investigate the effects of ceritinib on osteoclast formation *in vitro* and *in vivo*. We demonstrated that ceritinib also has a beneficial effect in preventing bone loss through the inhibition of osteoclast formation. Our findings suggest that ceritinib can not only suppress ALK-rearranged non-small cell lung cancer but also prevent osteoclastic osteolysis.

## Methods

### Cell proliferation assay (CCK-8)

The cell counting kit-8 (CCK-8, Dojindo, Japan) was used to examine the proliferation of BMMs. Cells were seeded into 96-well plates at a density of 3 × 10^3^ cells per well and cultured at 37°C with 5% CO2 for 12 h to allow the cells to adhere. The original medium was replaced with 10% (v/v) CCK-8/complete high glucose DMEM or α-MEM, and the cells were incubated for an additional 2 h. The Infinite M200Pro microplate reader (Tecan Trading AG, Hombrechtikon, Switzerland) was used to measure the absorbance at 450 nm. Each experiment consisted of three individual replicates.

### Osteoclast differentiation assay and bone resorption assay

Primary bone marrow-derived cells were extracted from the bone marrow of hindlimbs of 6 to 8-week-old C57BL/6J mice. After culturing in complete α-MEM (containing 10% FBS and 1% penicillin/streptomycin) in the presence of M-CSF (50 ng/ml, R&D Systems, MN, USA) for 4 days, all attached cells were treated as osteoclast precursor cells (bone marrow-derived monocytes and macrophages, BMMs), which were maintained and used for the following experiments. BMMs were subsequently induced to differentiate, using M-CSF (50 ng/ml) and RANKL (100 ng/ml, R&D Systems, MN, USA), for 5 days.

M-CSF-dependent BMMs were seeded into 96-well plates at a density of 1 × 104 cells/well in complete α-MEM and stimulated with 100 ng/ml of RANKL, as well as with different doses of ceritinib (CAS No.: 1032900-25-6; Cat. No.: HY-15656; purity: 99.98%; MedChemExpress LLC, China). The medium containing RANKL and the drug treatments were changed every two days. After 5-6 days of incubation, when large multinucleated osteoclasts were observed in the RANKL-only positive control group, all cells were fixed with 4% paraformaldehyde (PFA) and stained to detect tartrate-resistant acid phosphatase (TRAP). The TRAP-positive (violet) cells of more than or equal to 3 nuclei were defined as multinucleated cells (MNCs). The number, total area or area per cell were circled out manually, and quantified within a well as a whole, using the open-source software Fiji. Each set of experiments was repeated trice.

Then for bone resorption assay, M-CSF-dependent BMMs were also seeded in hydroxyapatite-coated Osteo Assay Surface Polystyrene Microplates (Corning Inc., NY, United States) at a density of 1.3 × 104 cells/well in complete α-MEM and stimulated with 100 ng/ml RANKL and respective agents for 7 days. Thereafter, the wells were incubated in 5% sodium hypochlorite solution to remove the cells. The bone resorption pits on the microplates were captured using a phase-contrast inverted light microscope (IX71; Olympus, Hamburg, Germany).

### RNA extraction and qPCR analysis of osteoclastogenesis-related genes

Total RNA from BMMs stimulated with RANKL in the presence or absence of ceritinib for 5 days, was extracted using the RNA miniprep kit (Axygen, AZ, USA) according to the manufacturer’s protocol. Then, cDNA was synthesized from total RNA using the PrimeScript RT Master Mix (Perfect Real Time) cDNA synthesis kit (RR036, Takara Bio Inc., Dalian, China) according to the manufacturer’s instructions. RT-qPCR was performed on the ABI Flex 6 real-time PCR System (Applied Biosystems, CA, USA) using the TB Green^®^ Premix Ex Taq™ (Tli RNaseH Plus) (RR420, Takara Bio Inc., Dalian, China). Each reaction was performed in triplicate. Primer sequences of osteoclastogenesis-related genes are listed as follows: (1) *Ctsk* forward: 5’ TAGCCACGCTTCCTATCCGA ‘3, reverse: 5’ CCTCCGGAGACAGAGCAAAG ‘3; (2) *Nfatc1* forward: 5’ TGTTCCTGGCAATAGCGTGT ‘3, reverse: 5’ AGGGTCGAGGTGACACTAGG ‘3; (3) *Acp5* forward: 5’ CCTCCGGAGACAGAGCAAAG ‘3, reverse: 5’ CATCGTCTGCACGGTTCTG ‘3; (4) *Gapdh* forward: 5’ ACCCAGAAGACTGTGGATGG ‘3, reverse: 5’ CACATTGGGGGTAGGAACAC ‘3. Relative fold changes in gene expression were calculated using the comparative CT (2^−ΔΔCT^) method.

### Western blotting

BMMs were pre-stimulated with ceritinib for 2 h in serum-free medium and then stimulated with RANKL (100 ng/ml) for the indicated time points. Total proteins from whole cells were extracted with RIPA Lysis Buffer (medium-level intensity) (Beyotime, Shanghai, China) in the presence of PMSF and protease inhibitor cocktail; subsequently, these proteins were electrophoretically separated using ExpressPlus PAGE (GenScript Laboratories, Piscataway, NJ, USA) and transferred onto PVDF membranes using an e-blot device (GenScript Laboratories, Piscataway, NJ, USA). The membranes were blocked with 5% (w/v) skim milk in tris-buffered saline (TBS) containing 0.1% (v/v) tween-20 (TBST, pH 7.4), and incubated for 1 h at room temperature (RT). The membranes were washed three times for 15 min with TBST and incubated for 12 h at 4°C with the following primary antibodies: p-Akt, Cell Signaling Technology #4060; Akt, Cell Signaling Technology #9272; p-p65, Cell Signaling Technology #3033; p65, Cell Signaling Technology #8242; p-ERK, Cell Signaling Technology #9101; ERK, Cell Signaling Technology #9101; and Actb, Cell Signaling Technology #3700. Membranes were washed thrice with TBST and incubated with either an IRDye 800CW anti-mouse secondary antibody (LI-COR) or an anti-rabbit secondary antibody conjugated to fluorescence (LI-COR), at 1:10000 dilution. Finally, the membranes were washed thrice with TBST and visualized using the Odyssey near-infrared (NIR) fluorescence imaging system (LI-COR, NE, USA). The grayscale ratio of each phospho-protein to its respective total protein was calculated. The ratios were then standardized, using the first control lane as the baseline (1.00), as shown below each western blot lane in the figures.

### Animal model

Female C57BL/6J (12-week-old) mice were purchased from Jihui (Shanghai, China) and maintained in an SPF-grade animal facility. Mice were anesthetized and subjected to ovariectomy (OVX) or sham operation. The mice were under observation for 1 week, and then 18 mice, in good condition, were randomly allocated into three groups (Sham, OVX, OVX plus ceritinib, n = 6 per group); mice in each group were intraperitoneally injected with PBS, PBS, and ceritinib (10 mg/kg body weight), respectively. The treatments were administered twice per week for a total of 8 weeks. At the end of the treatment period, all mice were euthanized and weighed. Hindlimb tissues were fixed in 4% PFA for 48 h. The tibiae were analyzed with micro-CT first and then decalcified in 10% EDTA (pH = 7.4) for 21 days.

Finally, all these tissues were embedded in paraffin and sliced for histological examination, including hematoxylin and eosin (H&E) staining and TRAP staining. TRAP staining results were evaluated based on two parameters: N. Oc (number of osteoclasts)/BS (bone surface) and Oc. S (osteoclast surface)/BS. All procedures were performed following the protocols approved by the Institutional Animal Care and Use Committee of Shanghai Ninth People’s Hospital, Shanghai Jiao Tong University School of Medicine.

### Micro-CT

Bone morphometry within the metaphyseal region of the proximal tibia was quantified with micro-CT (SkyScan 1176, Billerica, MA, USA) using an X-ray tube voltage of 50 kV and a current of 500 μA with a 0.5 mm aluminum filter. The resolution was set to 9 μm, and 1.8 mm of the bone sample was acquired for each CT scan. The 3D images of the scans were reconstructed and analyzed using the SkyScan NRecon program. Trabecular morphometry was characterized by measuring the bone volume fraction (BV/TV), trabecular thickness (Tb.Th), trabecular number (Tb.N), and trabecular spacing (Tb.Sp). Cortical morphometry was characterized by measuring the bone area (BA), tissue area (TA), cortical area fraction (BA/TA), and cortical thickness (Ct.Th).

### Statistical analyses

All data were analyzed with one-way ANOVA using GraphPad Prism 8 (GraphPad Software, San Diego, CA, USA). Once a significant effect was detected, a *post hoc* Student’s *t*-test was performed for further analysis. Results are presented as mean ± standard deviation (SD); number ≥ 3; all the *p-*values are displayed in the figures results; p < 0.05, p < 0.01, and p < 0.001 were considered statistically significant (*), highly statistically significant (**), and extremely statistically significant (***). To reduce the risk of type I errors, we considered |beta| ≥.20 as significant regarding the predictions.

## Results

### Ceritinib revealed no obvious cell toxicity in BMMs

**Figure 1:**
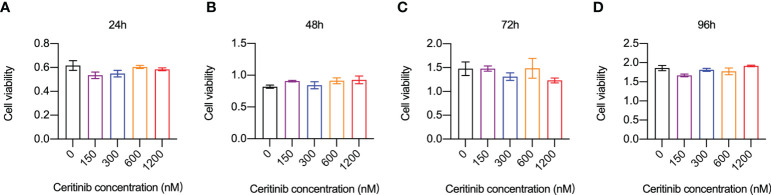
Ceritinib did not affect BMM-cell *in vitro*. Cell viability and proliferation capacities (absorbance) of BMMs remained stable when they were incubated with ceritinib (150, 300, 600, and 1200 nM) for 24 **(A)**, 48 **(B)**, 72 **(C)**, and 96 h **(D)**.

We examined whether the inhibitory effect of ceritinib on osteoclast formation was due to the inhibition of cell proliferation. Cell viability of BMMs was examined by incubating the cells with 150, 300, 600, or 1200 nM ceritinib. Compared to that in the control group, no obvious cell toxicity was detected in BMMs upon ceritinib treatment, indicating that ceritinib at concentrations of 150, 300, 600, or 1200 nM did not inhibit BMM proliferation until 96 hours (**Figure 1**).

### Ceritinib inhibited osteoclastogenesis inhibited osteoclast-specific gene expression *in vitro*

**Figure 2:**
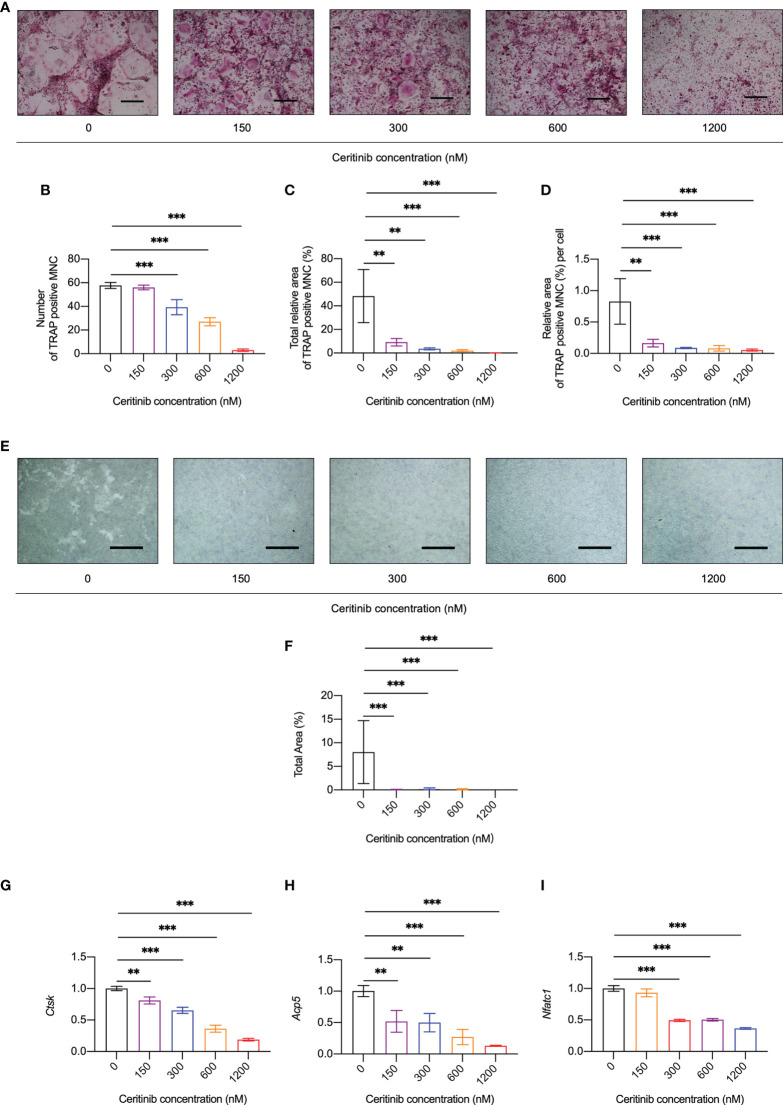
Ceritinib inhibited osteoclast differentiation and osteoclast-specific gene expression *in vitro*. **(A)** TRAP staining displayed diminishing tendency of osteoclast differentiation upon treatment with ceritinib (150, 300, 600, and 1200 nM). Scale bar = 10 µm. Statistical graphs show significantly reduced numbers **(B)** as well as decreased total **(C)** or per cell **(D)** area (%) of TRAP-positive MNCs upon treatment with different doses of ceritinib. **(E)** Bone resorption assay using hydroxyapatite-coated microplates showed a larger area of resorption pits after treatment with ceritinib (150, 300, 600, and 1200 nM). Scale bar = 200 μm. **(F)** Statistical graphs show significantly reduced total resorption area (%) upon treatment with different relative doses of ceritinib. MNC, multinucleated cell. The mRNA expression levels of osteoclast differentiation-related genes, *Ctsk* **(G)**, *Nfatc1* **(H)**, *Acp5* **(I)**, decreased upon ceritinib treatment (150, 300, 600, and 1200 nM).

To investigate whether ceritinib has any effect on osteoclast formation, we cultured BMMs with different concentrations of ceritinib (**Figure 2A**). Increased number of TRAP-positive and multinucleated osteoclasts was observed in the control group (**Figure 2B**). However, fewer TRAP-positive osteoclasts were observed in the presence of 150, 300, 600 or 1200 nM ceritinib (**Figure 2B**). We noticed a dose-dependent inhibitory effect of ceritinib on osteoclast formation. There was a significant decline in the number (**Figure 2B**) and total area (**Figure 2C**) of TRAP-positive multinucleated cells (MNCs) upon ceritinib treatment. The osteoclasts were smaller in the presence of ceritinib (**Figure 2D**). Taken together, these data suggest that ceritinib inhibits osteoclast formation *in vitro*. Next, we examined the bone resorption activity of osteoclasts, the bone resorption pit areas were unobvious in the 150, 300, 600 and 1200 nM ceritinib group compared to the positive control group (**Figures 2E, F**).

In agreement with the suppression of osteoclast formation and resorption activity *in vitro*, the expression of osteoclast-specific genes, such as cathepsin K (*Ctsk)* (**Figure 2G**), acid phosphatase 5 *(Acp5)* (**Figure 2H**), and nuclear factor of activated T-cells, cytoplasmic 1 (*Nfatc1)* (**Figure 2I**), was significantly inhibited by ceritinib at concentrations above 150 nM. Together, these data confirmed the inhibitory effect of ceritinib on osteoclast-specific gene expression.

### Ceritinib suppressed the phosphorylation of Akt and p65

**Figure 3:**
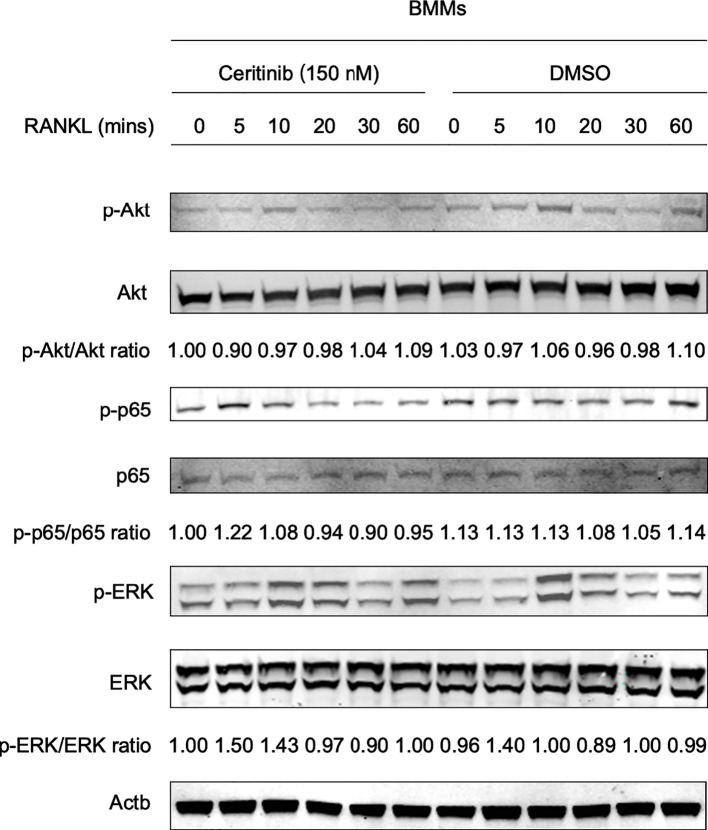
Molecular mechanisms of ceritinib in RANKL-mediated osteoclastogenesis. The status of different signaling pathways during RANKL-induced osteoclastogenesis: ceritinib inhibited the activation of phospho-Akt (Ser473) and phospho-p65. However, there was no significant change in ERK signaling.

Based on the observed inhibitory effect of ceritinib on osteoclast formation, we aimed to elucidate the possible underlying mechanisms. BMMs were treated with RANKL for 0, 5, 10, 20, 30, and 60 min in the presence or absence of ceritinib. As shown in **Figure 3**, RANKL activated Akt phosphorylation from 0 to 20 min. However, decreased Akt activation was observed in the ceritinib-treated group. In addition, phosphorylation of p65 was observed in the RANKL-treated group. However, ceritinib almost completely abolished the activation of p65. Interestingly, it did not alter the activation of the ERK signaling pathway (**Figure 3**). Collectively, ceritinib inhibited Akt and p65 phosphorylation during osteoclast formation.

### Ceritinib prevented ovariectomy-induced bone loss *in vivo*

**Figure 4:**
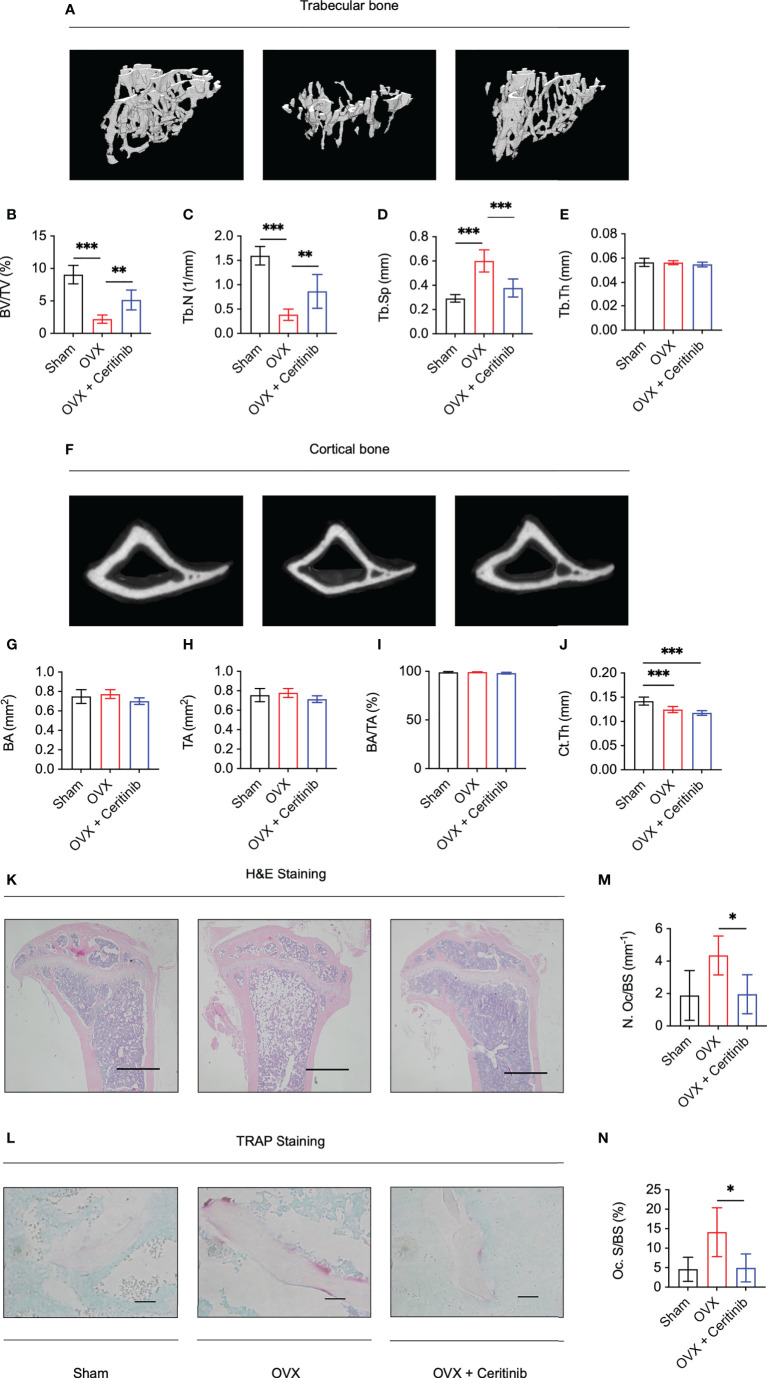
Ceritinib rescued osteoporosis by inhibiting osteoclasts *in vivo*. **(A)** Representative 3D micro-CT images of trabecular bone of the Sham, OVX, and OVX plus ceritinib groups. Micro-CT trabecular results suggest higher BV/TV **(B)** and Tb.N **(C)**, lower Tb.Sp **(D)**, and non-significant Tb.Th **(E)** in the OVX plus ceritinib group. **(F)** Representative 3D micro-CT images of cortical bone of the Sham, OVX, and OVX plus ceritinib groups. Micro-CT cortical results suggest no significant changes of BA **(G)**, TA **(H)**, BA/TA **(I)** and Ct.Th **(J)** in the OVX plus ceritinib group. **(K)** Representative images of H&E staining (Scale bar = 100 µm). **(L)** Representative images of TRAP staining (Scale bar = 2 µm). TRAP staining results demonstrate that ceritinib reduced the number **(M)** and decreased the surface of osteoclasts **(N)** per bone surface of trabecular bone compared to those in the OVX group.

Finally, to validate the inhibitory effect of ceritinib on osteoclast formation and function, we used an ovariectomy-induced osteoporosis model. Reconstructed images of the 3D scans of the left tibia are presented. There was a distinct decrease in trabecular and cortical bone volume in the OVX group, confirming the successful establishment of an ovariectomy animal model (**Figures 4A–J**). Compared with that in the OVX group of trabecular bone, higher BV/TV (**Figure 4B**) and Tb.N (**Figure 4C**), lower Tb.Sp (**Figure 4D**), and non-significant Tb.Th (**Figure 4E**) were observed in the OVX plus ceritinib group, suggesting that ceritinib prevented bone loss. Cortical bone results suggest no significant changes of BA (**Figure 4G**), TA (**Figure 4H**), BA/TA (**Figure 4I**), and Ct.Th (**Figure 4J**) in the OVX plus ceritinib group.

Further, histomorphometry analysis confirmed the preventive effect of ceritinib on bone loss. H&E staining confirmed the increased bone mass in the ceritinib-treated group compared to that in the OVX group. (**Figure 4K**). To assess the condition of osteoclasts *in vivo*, TRAP staining was performed, which showed fewer osteoclasts and decreased osteoclast surface per bone surface in the OVX plus ceritinib group compared to those in the OVX group (**Figures 4L–N**). Collectively, these data support the conclusion that ceritinib inhibited osteoclast formation in the OVX model.

## Discussion

Ceritinib is typically used to treat patients with ALK-rearranged non-small cell lung cancer. Our study demonstrated that, in addition to inhibiting cancer development, ceritinib was also beneficial in preventing bone loss. Our data showed that ceritinib effectively inhibited osteoclast formation and osteoclast-specific gene expression. More importantly, the inhibition of osteoclast formation resulted in the prevention of trabecular bone loss *in vivo*. Our result also suggested that ceritinib presented non-significant cortical bone loss prevention unlike the trabecular bone. According previous studies, cortical bone can be differentially affected by hormones and medications compared with trabecular one (30). Bone loss was first found in trabecular bone after menopause, and cortical bone later. The other explanation was that the effect of ceritinib on cortical bone probably need more time to observe. Notably, advanced non-small cell lung cancer metastasizing to the bone leads to osteolytic bone invasion (31–33). However, ceritinib shows great potential in repressing such skeletal-related events, besides suppressing tumor growth and extending survival.

ALK is a receptor tyrosine kinase that activates the PI3K-Akt signaling pathway (6). A previous study reported that overexpression of EML4-ALK variant 3 in HEK293T cells led to enhanced phosphorylation of Akt, whereas increased phosphorylation of ERK1/2 was not prominent in COS-7 cells (34). On the contrary, another study showed that treatment of NCI-H2228, a human non-small cell lung carcinoma cell line, with CH5424802 (a selective ALK inhibitor) led to reduction in p-Akt expression (35). Remarkably, crizotinib-resistant H3122CR-1 cells showed a dramatic downregulation of ALK and p-ALK and upregulation of the Akt/mTOR/S6 kinase pathways. Activation of autophagy in these cell lines positively altered the Akt/mTOR signaling pathway (36). The relationship between ALK and Akt indicated that ALK-driven neuroblastomas gradually acquired resistance to ALK inhibitors, but this effect was attenuated when combined with a p53 activator. This shift towards apoptosis, and away from cell-cycle arrest, is mediated by inhibition of the ALK–Akt–FOXO3a axis (37). In addition, previous studies have shown that nucleolar phosphoprotein nucleophosmin (NPM)/ALK, similar to other members of this family, activates PI3K and its downstream effector Akt (38, 39). This suggests that ceritinib, an ALK inhibitor, reduced the activity of ALK in order to negatively control the activity of Akt. In our study, we demonstrated that the underlying mechanism was due to the inhibition of phosphorylation of the Akt signaling pathways, which are canonical targets during osteoclastogenesis. Notably, Akt activates NF-κB signaling through phosphorylation of IκB (40, 41). A previous study suggested that in uterine carcinosarcomas, the ALK-mediated Akt/NF-κB/Twist1 pathway participates during the initial stage and regulates morphological alterations towards the sarcomatous phenotype (42). In addition, the induction of Akt was found to activate NF-κB/p65-dependent transcription, probably through repression of IκBα expression (43). Therefore, logically, the inhibition of p65 phosphorylation might be influenced by Akt inhibition in our study.

To the best of our knowledge, no previous study has reported that ceritinib can inhibit osteoclast formation and thus prevent bone loss. Interestingly, the first generation ALK inhibitor crizotinib has been reported to inhibit osteoclast formation and prevent prostate cancer bone destruction (29). These data suggest that ALK inhibitors have a preventive effect on bone loss. However, to generalize the applications in other metabolic bone diseases, the effect of ceritinib on other cells, such as osteoblasts, should also be taken into consideration. Also, to provide the stronger evidence to realistic bone invasion of cancer therapy, the administration of ceritinib on the mouse cancer models with bone loss (e.g., breast cancer, prostate cancer) should be analyzed in the future.

In conclusion, our data demonstrate that ceritinib can inhibit osteoclast formation by suppressing Akt and p65 phosphorylation, thereby preventing bone loss *in vivo*. Taken together, the use of ceritinib in patients with non-small cell lung cancer might improve their bone quality.

## Data availability statement

The original contributions presented in the study are included in the article/supplementary material. Further inquiries can be directed to the corresponding author.

## Ethics statement

This study was reviewed and approved by Institutional Animal Care and Use Committee of Shanghai Ninth People’s Hospital, Shanghai Jiao Tong University School of Medicine.

## Author contributions

Study Concept and Design: AQ. Data analysis: WH, XC, KK, KR, SH and AQ. Manuscript writing: WH and AQ. All authors contributed to the article and approved the submitted version.

## Funding

This work was supported by the Natural Science Foundation of China [Nos. 92068102, 81772373, and 81572167], Shanghai Municipal Education Commission - Gaofeng Clinical Medicine Grant Support, Shanghai Institute of Precision Medicine, Ninth People’s Hospital, Shanghai Jiao Tong University, Shanghai Jiao Tong University School of Medicine [the SHIPM-pi fund Nos. JY201804 and JC201801], Ninth People’s Hospital, Shanghai Jiao Tong University, Shanghai Jiao Tong University School of Medicine (No. JYJC202015], and the Foundation of National Facility for Translational Medicine (Shanghai) [No. TMSK-2020-119] to the corresponding author (Dr. An Qin).

## Conflict of interest

The authors declare that the research was conducted in the absence of any commercial or financial relationships that could be construed as a potential conflict of interest.

## Publisher’s note

All claims expressed in this article are solely those of the authors and do not necessarily represent those of their affiliated organizations, or those of the publisher, the editors and the reviewers. Any product that may be evaluated in this article, or claim that may be made by its manufacturer, is not guaranteed or endorsed by the publisher.
